# Anatomical Differences Determine Distribution of Adenovirus after Convection-Enhanced Delivery to the Rat Brain

**DOI:** 10.1371/journal.pone.0024396

**Published:** 2011-10-13

**Authors:** Sander Idema, Viola Caretti, Martine L. M. Lamfers, Victor W. van Beusechem, David P. Noske, W. Peter Vandertop, Clemens M. F. Dirven

**Affiliations:** 1 Neurosurgical Center Amsterdam and Cancer Center Amsterdam, Neuro-oncology Research Group, VU University Medical Center, Amsterdam, The Netherlands; 2 Section Oncogenomics, Department of Medical Oncology, VU University Medical Center, Amsterdam, The Netherlands; The University of Chicago, United States of America

## Abstract

**Background:**

Convection-enhanced delivery (CED) of adenoviruses offers the potential of widespread virus distribution in the brain. In CED, the volume of distribution (Vd) should be related to the volume of infusion (Vi) and not to dose, but when using adenoviruses contrasting results have been reported. As the characteristics of the infused tissue can affect convective delivery, this study was performed to determine the effects of the gray and white matter on CED of adenoviruses and similar sized super paramagnetic iron oxide nanoparticles (SPIO).

**Methodology/Principal Findings:**

We convected AdGFP, an adenovirus vector expressing Green Fluorescent Protein, a virus sized SPIO or trypan blue in the gray and white matter of the striatum and external capsule of Wistar rats and towards orthotopic infiltrative brain tumors. The resulting Vds were compared to Vi and transgene expression to SPIO distribution. Results show that in the striatum Vd is not determined by the Vi but by the infused virus dose, suggesting diffusion, active transport or receptor saturation rather than convection. Distribution of virus and SPIO in the white matter is partly volume dependent, which is probably caused by preferential fluid pathways from the external capsule to the surrounding gray matter, as demonstrated by co-infusing trypan blue. Distant tumors were reached using the white matter tracts but tumor penetration was limited.

**Conclusions/Significance:**

CED of adenoviruses in the rat brain and towards infiltrative tumors is feasible when regional anatomical differences are taken into account while SPIO infusion could be considered to validate proper catheter positioning and predict adenoviral distribution.

## Introduction

Convection-enhanced delivery (CED) is a novel way to circumvent the Blood-Brain Barrier (BBB) in the treatment of diseases of the brain including gliomas and neurodegenerative diseases [1]–[4]. Direct infusion of therapeutic agents into the interstitial space has the potential to achieve a widespread local distribution of the agent while minimising systemic degradation and toxicity. Ideally, the distribution volume (Vd) following CED is dependent on the infusion volume (Vi). Recent clinical trials have shown the safety of CED in the treatment of Glioblastoma Multiforme (GBM) [5]–[7]. In addition, these trials have illustrated some of the technical challenges of CED and the need to monitor the process of convection to prevent toxicity on one hand and frank ineffectiveness due to limited convection on the other [8]. Adenoviral vectors or conditionally replicating adenoviruses (CRAds) have been used preclinically and clinically in a variety of intracranial diseases [9], [10]. After intravascular, intrathecal or intracerebral delivery, adenovirus distribution and therapeutic effects are limited due to the presence of the BBB, limited diffusion of injected viral particles or elimination by the activated immune system [11]–[13]. As CED relies on bulk flow instead of diffusion, it represents a promising method to deliver adenoviruses to the brain. Thus far, controversy exists whether adenoviruses are suitable agents for CED [14], [15]. The size of the adenoviral particle (70–90 nm) is considered to be an important determinant of its distribution, although other factors have been suggested as well [14], [16].

Ideally, CED of adenovirus should be monitored by either directly visualizing the viral particle, or by infusion of a surrogate marker with similar CED-characteristics. To determine whether regional anatomical differences influence adenoviral distribution we convected an adenoviral vector expressing Green Fluorescent Protein (GFP) into the gray and white matter of the rat brain. Additionally, we infused Superparamagnetic Iron Oxide Nanoparticles (SPIOs) as a surrogate tracer to predict the volume of adenovirus distribution after CED, while proper infusate delivery was confirmed by co-infusion of trypan blue. Finally, infiltrative tumors were targeted using white matter tracts as the delivery route for adenoviruses and SPIO.

## Results

### Albumin does not affect in vitro adenovirus infection efficiency

Because most agents used for CED are stored and delivered in solutions containing albumin we first investigated if the albumin concentration would influence infection efficiency. To this end, we subjected U-251MG glioma cells for 30 minutes to AdGFP in medium containing various concentrations of human albumin. The percentage of infected cells ranged from 5.6±0.6% (no HSA) to 3.9±0.4% (0.4% HSA). Although higher concentrations (0.4% and 4%) of HSA slightly decreased infection efficiency, the differences were neither large nor significant (p>0.1 for all comparisons).

### Infusion of AdGFP into rat striate – gray matter

**Figure 1:**
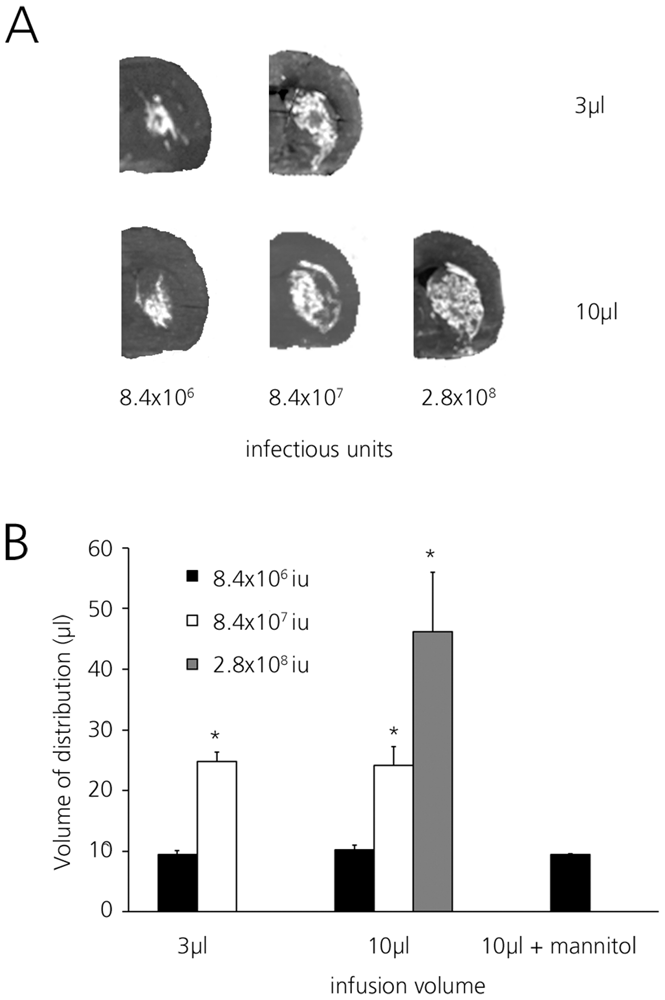
Volumes of distribution (Vd) of adenoviral transgene expression in rat striate. GFP expression following 3 µl (left side) or 10 µl infusion (right side) of AdGFP as detected by fluorescence imaging using IVIS lumina imaging system. Vd was calculated from serial slides by determining GFP expression. (A) Distribution after infusion of AdGFP in 3 µl (upper) or 10 µl (lower) at increasing dose as determined by IVIS fluorescence. Representative sections from rat hemispheres are shown. (B) Vd of AdGFP infused in either 3 µl or 10 µl with increasing dose. 8.4*10^6^ iu (black bars), 8.4*10^7^ iu (white bars) and 2.8*10^8^ iu (gray bars). Differences between 3 µl and 10 µl infusion volume at similar doses were not significant. Mannitol did not increase Vd. Average Vd ± SD is shown, * p<0.01 compared to lower dose.

AdGFP was infused in three different doses into the corpus striatum of Wistar rats in 3 µl or 10 µl total volume at a rate of 0.33 µl/minute (figure 1A). In all cases, the area of GFP expression was confined to the striate with minimal backflow occurring into the white matter of the external capsule.

At the two lower virus doses, the Vd achieved with the two infusion volumes (3 µl and 10 µl) was almost the same reaching approximately 10 µl at 8.4×10^6^ iu and approximately 25 µl at 8.4×10^7^ iu (figure 1B). Infusing the maximum dose that could be delivered (2.8×10^8^ iu in 10 µl) resulted in a further increase of Vd to 46.2±9.8 µl, almost filling the complete striate. These results demonstrate that the distribution of AdGFP in the rat striate is dependent on the dose, rather than the infusion volume.

In an attempt to increase the interstitial space, mannitol was co-infused but this did not change the resulting Vd (9.3±0.2 µl versus 10.2±0.7 µl, p>0.3; figure 1B).

### Infusion of AdGFP into corpus callosum and external capsule - white matter

**Figure 2:**
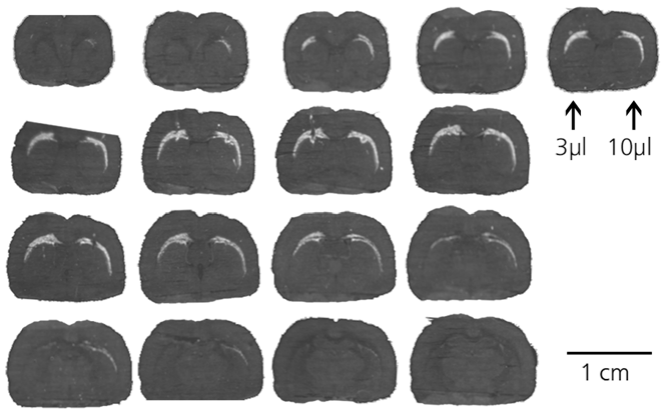
Distribution of adenoviral transgene expression in rat corpus callosum and external capsule. 3 µl or 10 µl of AdGFP containing 8.4*10^6^ iu was infused into the external capsule. 30 µm sections of one representative rat are shown. Left side of images: 3 µl infusion, right side: 10 µl infusion. Slides are arranged in rostrocaudal (from upper left to lower right) direction. GFP expression is extended when virus is infused in 10 µl compared to bolus, both in coronal and sagittal planes.

AdGFP was infused into the most prominent white matter tract of the rat (figure 2). In contrast to the striate, the typical anatomical orientation of the corpus callosum and the external capsule, thinning rapidly lateral from the corpus callosum, complicates objective volume measurements. Increasing the infusion volume from 3 µl to 10 µl increased the Vd insignificantly from 7.1±4.8 µl to 9±2.5 µl. More relevant however, the maximum distance traveled by the viral particles as shown by GFP expression following infusion in 10 µl was on average 1.4-fold greater than following infusion in 3 µl, both in the coronal (1.4±0.2, p<0.05) and the sagittal (1.4±0.1, p<0.05) plane. The maximum distance from the point of infusion traveled by the virus injected in 10 µl was 6.1 mm in the coronal plane and 2.4 mm in the sagittal plane. These results indicate that in the white matter, the distribution of adenovirus is at least partly dependent on infusion volume.

### Prediction of Adenoviral Vd by superparamagnetic iron oxide nanoparticle infusion

**Figure 3:**
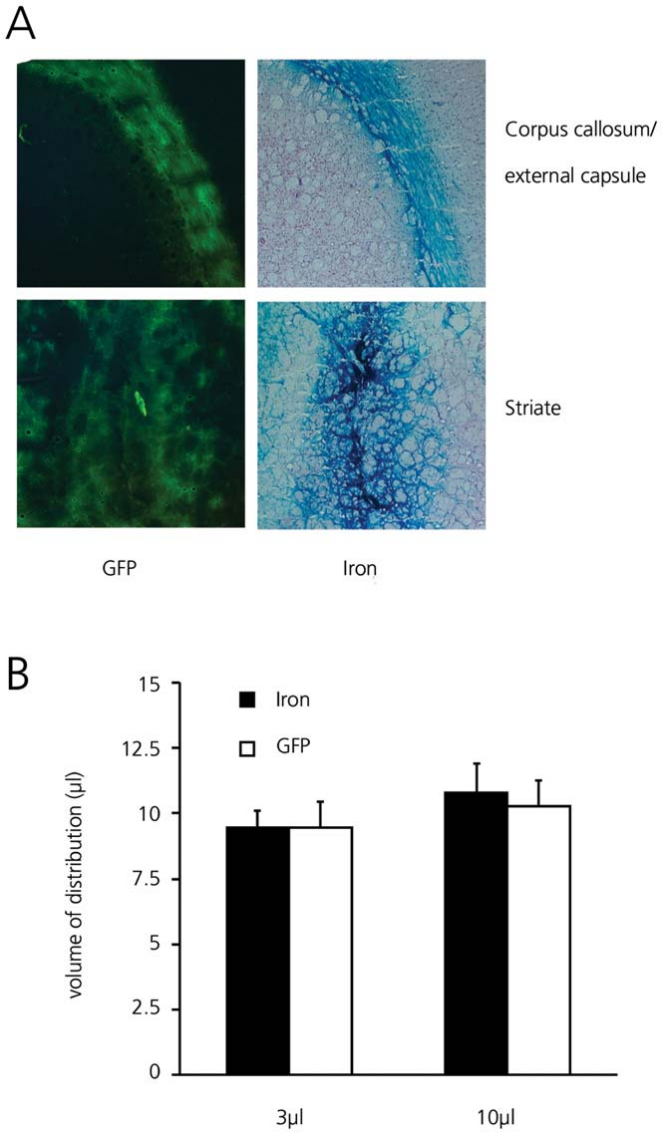
Co-infusion of SPIO and AdGFP in rat striate and corpus callosum. (A) Parallel 30 µm coronal sections three days after infusion. GFP and HE & iron stained sections showing GFP expression in external capsule and striate (left column) and corresponding iron distribution (right column). Original magnification 5×. (B) Vd of SPIO (black bars) and AdGFP (white bars) following 3 µl or 10 µl infusion into rat striate combined with AdGFP. No significant differences were noted. Average Vd ± SD are shown.

25 µg of SPIO and 8.4×10^6^ iu adenovirus were co-infused in either 3 µl or 10 µl into the rat striate or external capsule. Adenovirus Vds and distribution patterns were comparable to those found when Ads were infused without SPIO and the Vds of co-infused SPIO and virus were completely overlapping (figure 3A). In the striate, there was no increase in distribution with increasing infusion volume, while in the corpus callosum and external capsule viral spreading, as measured by the distance covered by GFP expression, increased with increasing Vi. SPIO Vds in the striate were 9.4±0.7 µl and 10.8±1.1 µl following 3 µl and 10 µl infusion, respectively, comparable to the Vd of GFP (figure 3B). In the white matter a similar relation between covered distance and infusion volume was found for SPIO and adenovirus (including a factor 1.4 difference between 3 µl and 10 µl). From these results it appears that 64 nm SPIO and adenovirus distribute in a similar fashion through the rat brain.

### Glial cell infection by adenovirus

**Figure 4:**
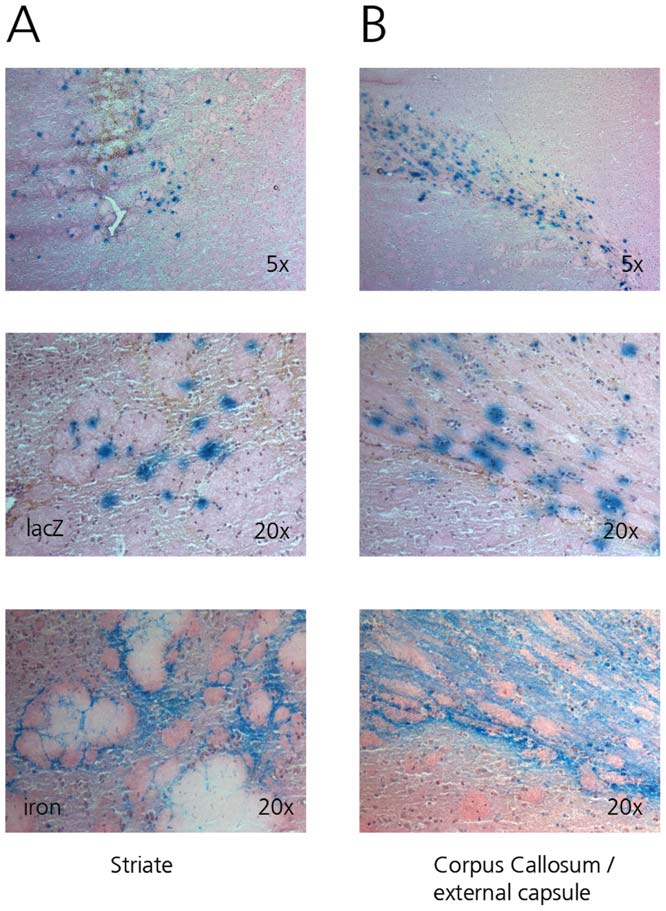
Determination of infected cells by nuclear morphology (top 2 rows, original magnification 5× and 20×, HE staining). β-Gal staining shows glial cells are infected following infusion of AdCMVLacZ in the rat striate (A) or external capsule (B). No infected neurons were detected. No infected cells were detected in the overlying neocortex. SPIO distribution (iron staining) is similar to β-Galactosidase staining both in gray and white matter (bottom row, original magnification 20×).

Axonal transport of GFP, especially in the white matter, could theoretically have biased the determination of the actual Vd. As GFP is not the most sensitive marker for the determination of the type of infected cells, 8.4×10^6^ iu of Ad.LacZ was infused together with 25 µg SPIO in 10 µl PBS. β-galactosidase is expressed from this vector in the nuclei of infected cells, allowing for a more specific determination of the type of infected cells by nuclear morphology. Infected cells were primarily oligodendrocytes, astrocytes and other glial cell types (figure 4). No infected neurons were observed and no β-galactosidase positive cells were seen in the overlying cortex, confirming that the GFP expression seen was not due to axonal transport, but probably caused by GFP expression in the myelin sheath forming glial cells. Therefore adenovirus appeared to infect similar glial cell types in both white and gray matter. Co-infused SPIO co-localized with infected nuclei in both locations.

### Co-infusion of SPIO and trypan blue

**Figure 5:**
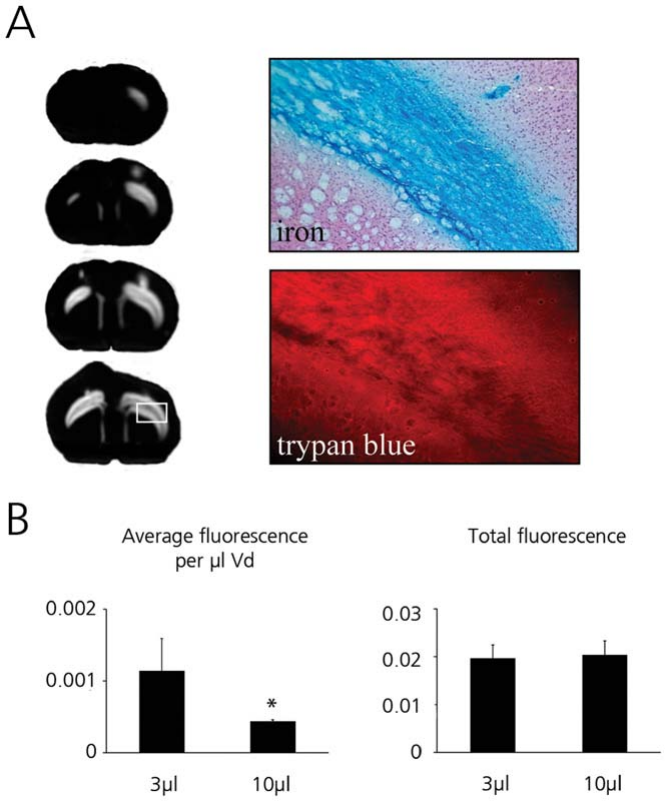
Determination of fluorescence of trypan blue and SPIO distribution following CED into the external capsule. Trypan blue fluorescence was detected using the IVIS Lumina system and by fluorescent microscopy. Fluorescence was quantified using ImageJ software. (A) Trypan blue is detected in the gray matter surrounding the corpus callosum, while SPIO is confined to the corpus callosum. Trypan blue is easily detected using fluorescent imaging. Both IVIS imaging (left) and microscopy fluorescence imaging (lower right panel, showing magnified view of the area marked white in left panel) yield similar distribution patterns. Image shows coronal sections through rat brain. Fluorescence signal showing in white. Right side 10 µl infusion, left side 3 µl infusion. Upper right panel shows corresponding HE & iron staining in parallel section, showing SPIO distribution confined to the corpus callosum and external capsule (original magnification 5×). (B) Trypan blue fluorescence used to determine if CED was successful. Average fluorescence per µl distribution volume is decreased on the 10 µl side due to the larger Vd. Total fluorescence in both groups is similar indicating equal delivered dose. Average fluorescence ± SD is shown.

Adenovirus and SPIO distribution in the white matter increased with increasing volume, but not as much as would be predicted on the basis of the volume of the interstitial space (which would yield a Vd/Vi ratio of 5). We hypothesized that the effect of CED of viral particles in rat white matter might be limited by infusion fluid partly entering the gray matter, while larger particles distributed only in the white matter. To investigate this, SPIO were co-infused with trypan blue into the external capsule and rats were immediately sacrificed after infusion. As before, SPIO distributed only in the white matter of the corpus callosum and the external capsule. In contrast, trypan blue could be detected in the white matter and extending into the gray matter of both overlying neocortex and corpus striatum (figure 5A). In these particular experiments, total Vds of SPIO were 8.9±6 µl (Vi = 3 µl, Vd/Vi ratio 3.0) and 16.9±4 µl (Vi = 10 µl, Vd/Vi ratio 1.7). The corresponding trypan blue Vds were 18.4±5 µl (Vd/Vi ratio 6.1) and 47.1±3 µl (Vd/Vi ratio 4.7), showing effective trypan blue convection with a high Vd/Vi ratio close to the predicted value. This was confirmed by measuring the total fluorescence signal and average fluorescence per unit volume (figure 5B). While similar amounts of trypan blue were delivered (measured by total fluorescence), infusion in a larger volume yielded a lower fluorescence per volume due to distribution over a larger volume.

### Targeting infiltrative tumors in the white matter

C6 infiltrative brain tumors located in the external capsule were targeted via white matter tracts using a mixture of SPIO and trypan blue or SPIO, AdGFP and AdΔ24-CMV-GFP. The latter mixture was chosen as *in vitro* results showed that the replicating AdΔ24-CMV-GFP at 100 iu/cell provided almost a 1000-fold higher transgene expression in C6 cells when compared to AdGFP (89.7±5.3% vs. 0.1±0.6% transduction, respectively, p<0.0001), while AdGFP showed higher levels of transgene expression in normal brain (data not shown).

**Figure 6:**
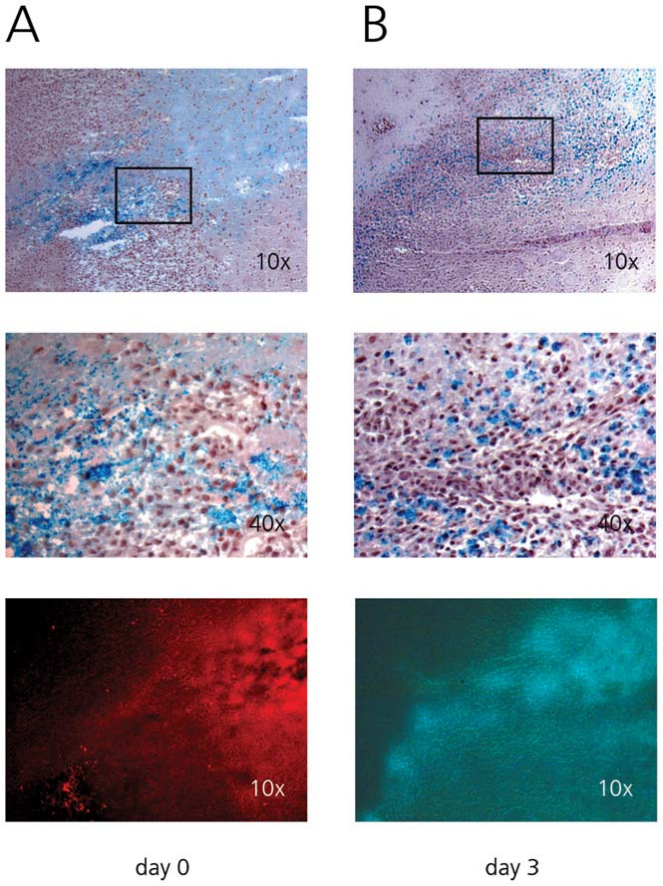
CED of trypan blue, SPIO and adenovirus targeting infiltrative C6 tumors. When tumor infiltrated the white matter, iron particles are seen engulfing tumor cells. Penetration into solid tumor is limited either with SPIO (top two rows), trypan blue (A, bottom row) or adenovirus infusion (B, bottom row). Bottom row trypan blue and GFP images correspond to top row Perls stained images. (A) Iron and trypan blue distribution directly after infusion. (B) Iron and GFP distribution three days after infusion.

Although tumor and infusion locations were several millimeters apart, directly after infusion SPIO was noted at locations where tumor cells infiltrated the white matter tracts (figure 6A). Neither iron particles nor trypan blue showed efficient penetration into solid tumor. Iron particles were noted surrounding single tumor cells, appearing to be extracellular at this stage. Three days after infusion of SPIO and adenovirus, GFP expression was seen at the rim of tumors, co-localizing with iron distribution within the solid tumor (figure 6B). Iron particles now appeared to be intracellular although no differentiation between uptake in tumor cells and reactive astrocytes could be made. Tumors that were located only just outside the white matter in the cortex were not transduced (data not shown).

## Discussion

In the current study we used a small diameter fused silica infusion cannula to convect an adenoviral vector into the rat brain and towards brain tumors. We show that while the volume of distribution is related to infused dose in the gray matter of the corpus striatum, it appears to be related to infusion volume in the white matter of the corpus callosum and external capsule. In addition we show that the distribution of Ads is similar to that of similar sized SPIO that can thus be used to predict the distribution of adenoviruses in the brain. Finally, distant tumors can be reached by CED of adenoviruses, but penetration of these tumors is limited.

The results have some important implications for the delivery of adenoviruses in the treatment of neurologic diseases for which they have recently been used and especially for brain tumors where the migrated cells in the surrounding brain parenchyma, mainly consisting of white matter, are the target of treatment [9], [12], [17].

Because the albumin concentration of the infusion fluid had no influence on infection efficiency, a 4% albumin concentration was chosen as this concentration has been shown to increase the distribution volume of adenoviruses [16], possibly because albumin stabilizes the viral solution and prevents aggregation of viral particles [18], which could affect their distribution.

Ideally, CED of adenoviruses in a clinical setting should rely on the infused volume instead of the infused dose, as this would permit control over the desired distribution volume. Most of the recent studies using adenoviral vectors in the brain did not investigate the effect of infusion volume on the distribution of adenoviruses [16], [19]–[21]. One previous study that did, reported a correlation to infused dose rather than infused volume when an adenoviral vector was infused into the rat striate [14], which is similar to the results obtained from the striatal gray matter infusions in our study and other studies infusing large particles in the striate [22], [23]. These results suggest that sufficient room exists in the interstitial space of the striate for viral particles to distribute and that this distribution is not dependent upon convection, but more likely on other factors such as receptor saturation or diffusion. Because the width, or pore size, of gray matter extracellular space is estimated to be 38–64 nm [24], it appears likely that adenovirus distribution in the striate does not occur in the parenchymal interstitium but along preferred routes such as perivascular spaces which have a far greater diameter (500 nm) [25]. This distribution pattern has previously been observed with other large particles such as liposomes and adeno-associated viruses and is thought to be dependent on cardiac pulsations that propel the particles in the perivascular space [26]–[28]. The observation in our study that the Vd/Vi ratio could increase well beyond the expected value strongly suggests that such an active transport also affects adenoviral distribution and could obscure any effects from infusion driven bulk flow. In addition, the lack of an increased Vd when mannitol is co-infused to extend the interstitial space supports the concept of perivascular propagation because the perivascular spaces have sufficient space to accommodate viral transport, even without the addition of mannitol.

In the white matter of the corpus callosum and external capsule, the distribution of adenoviruses appears to be dependent on infusion volume. This was however not reflected in a substantially increased Vd. Most likely the adenoviral particle is confined to the white matter whereas the infusion fluid is not, which we showed in the experiments co-infusing trypan blue and SPIOs with the latter exhibiting a similar distribution pattern as adenovirus. A possible explanation for the differential distribution of adenoviruses in white compared to gray matter could be related to differences in ECS composition. Both electron microscopy and mathematical analysis have shown that the white, but not the gray, matter ECS is easily extended by edema or infusion fluid [29]–[31], possibly enough to allow the transport of adenovirus by bulk flow. In addition, glypican, a member of the heparan sulfate proteoglycans that are known to act as co-receptors for adenovirus binding [32] is expressed in gray but not white matter of the rat [33]. Increased adenovirus binding to glypican in gray matter could also explain the limited propagation of virus particles in gray matter. Interestingly, in a study infusing Indian ink, it has been shown that this large particle (250 nm) spreads diffusively through the interstitial space of white matter but was confined to the perivascular spaces in gray matter [34]. Assuming a similar distribution pattern of adenoviruses would explain why the viral particle is distributed by Vi dependent interstitial bulk flow in the white matter and by perivascular transport in the striatal gray matter.

Differential expression of transgene by neurons and glial cells could partly explain the noted transgene distributions, as neuronal cell bodies will be more abundant in gray matter. We observed no differences in transduced cell types between white and gray matter, showing only transgene expression in glial cells. Obviously, these results do not rule out the infection of neurons by adenovirus that has been observed by others [20], [35] as transgene expression may have been below detection limits. However, such a differential expression would not likely explain the differences in distribution volume when only the infusion volume is changed.

The scarcity of white matter in the rat brain compared to the abundance in the human brain raises issues on the use of this animal as a model for CED. From the current study we cannot determine if the relation between Vi and Vd in white matter is maintained when larger volumes are infused. Indeed, the larger canine brain may be better suited for preclinical evaluation of this technique [19], [20], [35]. In addition, the use of such a model would provide the possibility to study the distribution of these particles in other white matter tracts that may be clinically relevant.

Distribution patterns of SPIOs were comparable to those of adenovirus. The SPIO used, Resovist®, has a hydrodynamic diameter of 64 nm, which makes it comparable, though slightly smaller, to adenovirus. Iron particles have been used to predict the distribution of adeno associated viruses (AAV) by MRI [23]. From the results of our experiments it appears that distribution of adenovirus and SPIO is very similar and since MR imaging of these agents has been shown to be feasible at a concentration of 1 mg/ml [22], [23], [36], which is slightly less than the concentration used in this study, SPIO infusion could thus be considered to confirm the proper positioning of CED catheters. This strategy could be used to prevent adenoviral distribution to undesired locations and to predict the distribution of adenoviruses following CED.

When tumors located in the white matter were targeted by infusing adenoviruses and SPIO from a distant location, we show that while the particles reach and engulf infiltrating tumor cells, infection of tumor cells or deposition of iron particles is confined to the outer rim of the solid tumor parts. Even trypan blue does not easily penetrate the solid tumor parts. In addition, when tumors were located only slightly outside the main white matter tract, no transduction of tumor cells was noticed. Factors explaining these results may be the high interstitial pressure in solid tumors and the increased tortuosity of brain tumor extracellular space limiting diffusion of particles [37]–[39]. In the specific case of gene therapy, these results imply that transduction of distant solid tumors using non replicating vectors will prove to be cumbersome, but that this technique might be used to reach solitary migrating tumor cells. In addition, CED of replicating (oncolytic) adenoviruses might be employed to provide a starting point for adenoviral oncolysis following infection of the rim of solid tumor parts. Care should be taken when using the C6 model in combination with viruses as the allogeneity of this model might elicit strong immune responses, even without the addition of viruses [40].

In CED, catheter placement is crucial. Our results suggest that placement of catheters in the gray matter should be avoided when convecting large particles such as adenoviruses. In addition we show that CED of adenoviruses is incapable of delivering viral particles in the gray matter using white matter tracts. These factors should be considered when catheter planning is performed and could possibly be integrated into models predicting convective delivery [41].

In conclusion, the current study shows that CED of Adenoviruses is feasible when gray and white matter differences are considered and the distribution can be predicted by SPIO infusion. The observed distribution pattern of adenovirus after CED closely mimics the routes of migrating single tumor cells [42], [43], which could make CED of adenoviruses a suitable delivery method to track these cells in the treatment of gliomas.

## Materials and Methods

### Ethics statement

All animal experiments were performed according to the guidelines established by the European community and following a protocol approved by the local ethical and scientific committees on animal experiments at our institution.

### Tumor cells and Adenoviral vectors

U-251MG malignant glioma cells and C6 rat glioma cells were obtained from the ATCC (Manassas, VA) and maintained in DMEM containing 10% Fetal Calf Serum (Gibco BRL Life Technologies, Paisley, UK).

AdGFP and AdCMVLacZ are replication-deficient adenoviral vectors carrying an expression cassette for enhanced green fluorescent protein and the Escherichia coli β-galactosidase gene respectively under control of the human CMV promoter in the deleted E1 region of the adenoviral backbone and have been described before [44], [45]. Vectors were propagated on 293 cells and purified by CsCl gradient according to standard techniques. AdΔ24-CMV-GFP was constructed by inserting a GFP expression cassette under control of the CMV promoter in the deleted E3 region of AdΔ24 [46], which carries a 24 bp deletion in the E1a region, limiting replication to cells with a dysfunctional Rb pathway [47]. AdΔ24-CMV-GFP was plaque purified and propagated on A549 cells. Viral titers in infectious units (iu) were determined by limiting dilution assay on 911 cells by hexon protein staining 48 hours after infection.

### Infection efficiency assay

U-251MG cells were plated in 96-well plates at 10,000 cells per well. The next day, cells were washed in PBS and AdGFP was added at 5 iu/cell for 30 minutes with human serum albumin (HSA, Cealb, Sanquin, Amsterdam, the Netherlands) diluted in serum-free DMEM. HSA concentrations were 0% (control), 0.04% (approximate CSF albumin concentration) 0.4% (approximate concentration in cell culture medium containing 10% FCS) and 4% (albumin concentration in normal human serum). After this period, cells were washed three times in PBS, and fresh complete culture medium was added. GFP expression was assessed after 36 hours by FACS analysis. The infection efficiency of C6 cells was assessed by adding either AdGFP or AdΔ24-CMV-GFP for one hour at 100 iu/cell and determining the percentage of cells expressing GFP after 36 hours.

### Infusion system

Pressure from a syringe pump (PHD 2000, Harvard Apparatus Inc., Holliston, MA) was transmitted to a syringe by way of a non-compliant and zero dead volume pressure transduction system described by Lonser et al. [48] Infusate was loaded into a 10 µl Hamilton gastight syringe (RN1701, Hamilton Comp., Reno, NV) attached at one end to the pressure transduction system and on the other side to a 168 µm outer diameter fused silica cannula. This cannula was chosen as it showed minimal backflow in preliminary experiments using an infusion rate of 0.33 µl/minute.

### Animal experiments

Female Wistar rats weighing 250–300 grams (Harlan, Horst, the Netherlands) were anesthetized by an intraperitoneal injection of 90 mg/kg ketamine and 10 mg/kg xylazine. The heads were shaved and the rats were placed in a stereotactic frame (Stoelting co., Wood Dale, IL). A midline incision was made to expose the cranium where two 0.8 mm burr holes were made on either side 0.5 mm anterior and 3.5 mm lateral from the bregma. The tip of the infusion cannula was lowered into the corpus callosum/external capsule (3.5 mm) or caudate putamen (6 mm) of the rat. 3 µl or 10 µl of 4% HSA in PBS containing the indicated amounts of AdGFP, AdCMVLacZ, 25 µg Resovist® (SPIO, ferucarbotran, 64 nm hydrodynamic diameter, Schering, Berlin, Germany), 1 µl 0.4% trypan blue (molecular weight 960, Sigma, St. Louis, MO), or a combination of these were infused at a rate of 0.33 µl/minute. To increase the extracellular space fraction, one group of rats received AdGFP in 10 µl of a solution containing 4.2% mannitol. Five minutes after the end of infusion the cannula was removed at a rate of 0.5 mm/minute. Three to four rats per group were used.

In the tumor experiments 500,000 C6 glioma cells were injected in a volume of 3 µl PBS 5.5 mm lateral and 0.5 mm anterior to the bregma at a depth of 6 mm in the external capsule. Tumors were allowed to grow for 4 days. After this period, a mixture of SPIO and trypan blue or SPIO, AdGFP and AdΔ24-CMV-GFP (10 µl at 0.33 µl/minute) was infused in the external capsule (bregma +0.5 mm, lateral 3.5 mm, 3.5 mm deep).

Rats that were injected with adenovirus were sacrificed after 3 days, whereas rats that were injected with trypan blue were sacrificed directly at the end of infusion. Sacrificing the rats was performed by i.p. pentobarbital overdose (>120 mg/kg). Brains were removed and snap frozen in liquid nitrogen for histochemistry and determination of GFP and β-galactosidase expression, iron and trypan blue distribution.

### Determination of volumes of distribution and histochemistry

Rat brains were cut into 8 µm or 30 µm sections and slides were assessed for GFP expression (green filter, 445–490 nm EX/515–575 nm EM) or trypan blue fluorescence (far-red filter, 615–665 nm EX/695–770 nm EM) by imaging using the IVIS Lumina imaging system (Xenogen, Cranbury, NJ). The threshold level of GFP expression was determined to be 10% of peak GFP expression. The area of trypan blue staining was determined by the observed area of fluorescence in ImageJ with 10% of peak fluorescence set as threshold. β-Gal staining was performed according to the manufacturer's instructions (β-Gal Staining Set; Boehringer Mannheim, Almere, The Netherlands).

Iron particles were detected by imaging of Perls stained sections. Perls iron stain was done by incubating in equal parts of 2% HCl and 2% potassium ferrocyanide yielding a blue reaction product. Slides were counterstained using hematoxylin and eosin.

Volumes of distribution were determined by multiplying the calculated areas of distribution in serial slides with the interslide distances (300 µm).

### Statistical analysis

Differences in infection efficiency and volumes of distribution were compared by two-sided t-test. In all experiments, p<0.05 was considered statistically significant.
